# CellCommuNet: an atlas of cell–cell communication networks from single-cell RNA sequencing of human and mouse tissues in normal and disease states

**DOI:** 10.1093/nar/gkad906

**Published:** 2023-10-18

**Authors:** Qinfeng Ma, Qiang Li, Xiao Zheng, Jianbo Pan

**Affiliations:** Precision Medicine Center, The Second Affiliated Hospital of Chongqing Medical University, Chongqing 400010, China; Basic Medicine Research and Innovation Center for Novel Target and Therapeutic Intervention, Ministry of Education, Institute of Life Sciences, Chongqing Medical University, Chongqing 400016, China; Basic Medicine Research and Innovation Center for Novel Target and Therapeutic Intervention, Ministry of Education, Institute of Life Sciences, Chongqing Medical University, Chongqing 400016, China; Basic Medicine Research and Innovation Center for Novel Target and Therapeutic Intervention, Ministry of Education, Institute of Life Sciences, Chongqing Medical University, Chongqing 400016, China; Precision Medicine Center, The Second Affiliated Hospital of Chongqing Medical University, Chongqing 400010, China; Basic Medicine Research and Innovation Center for Novel Target and Therapeutic Intervention, Ministry of Education, Institute of Life Sciences, Chongqing Medical University, Chongqing 400016, China

## Abstract

Cell−cell communication, as a basic feature of multicellular organisms, is crucial for maintaining the biological functions and microenvironmental homeostasis of cells, organs, and whole organisms. Alterations in cell−cell communication contribute to many diseases, including cancers. Single-cell RNA sequencing (scRNA-seq) provides a powerful method for studying cell−cell communication by enabling the analysis of ligand−receptor interactions. Here, we introduce CellCommuNet (http://www.inbirg.com/cellcommunet/), a comprehensive data resource for exploring cell−cell communication networks in scRNA-seq data from human and mouse tissues in normal and disease states. CellCommuNet currently includes 376 single datasets from multiple sources, and 118 comparison datasets between disease and normal samples originating from the same study. CellCommuNet provides information on the strength of communication between cells and related signalling pathways and facilitates the exploration of differences in cell−cell communication between healthy and disease states. Users can also search for specific signalling pathways, ligand−receptor pairs, and cell types of interest. CellCommuNet provides interactive graphics illustrating cell−cell communication in different states, enabling differential analysis of communication strength between disease and control samples. This comprehensive database aims to be a valuable resource for biologists studying cell−cell communication networks.

## Graphical Abstract

**Graphical Abstract:**
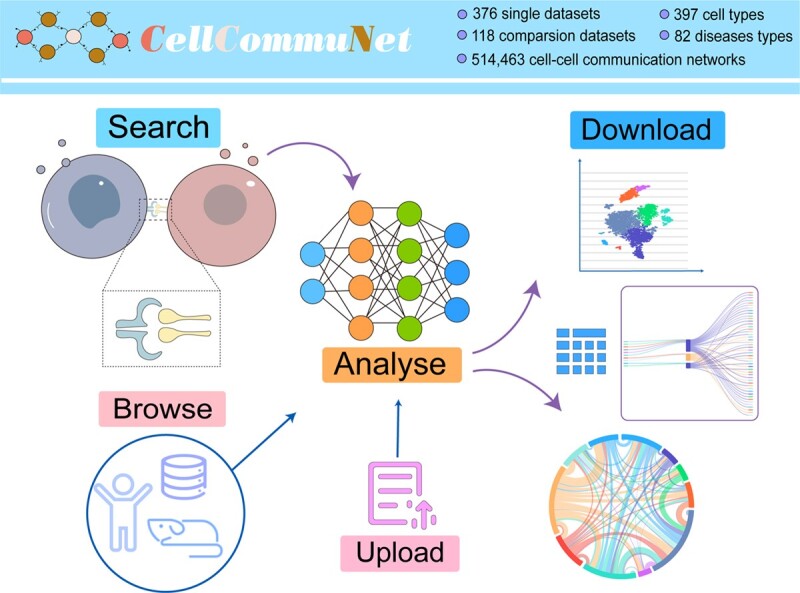

## Introduction

The biological activities of multicellular organisms rely on the collaboration of cells within the body, which in turn depends on communication among various cells (1,2). The dynamic network of intercellular communication and cooperation is crucial for maintaining the biological functions of cells and organs and microenvironmental homeostasis. Disruption or abnormalities in cell communication can lead to functional disorders and, in severe cases, to the development of diseases and even cancers. For example, the interactions between macrophages and erythropoietic cells in erythropoietic islands contribute to normal erythropoiesis (3), while there is a significant correlation between the occurrence of specific ligand−receptor interactions and the extent of regulatory T-cell infiltration and tumour growth (4). Normal cell communication mainly relies on a range of molecules present in the body, such as ligands, receptors, ions, hormones, neurotransmitters, and cytokines. Traditional research has often focused on studying specific cell types and signalling molecules through *in vitro* experiments using techniques such as yeast two-hybrid screening and coimmunoprecipitation (5). While these methods can accurately measure the interactions between specific cell types, they neglect the fact that cell communication occurs in the context of multiple cell types and numerous signalling molecules. With the rise of single-cell technologies, researchers can obtain gene expression profiles at single-cell resolution, allowing a more comprehensive exploration of cell−cell communication networks (6). Additionally, single-cell technologies offer advantages in identifying cellular heterogeneity within complex tissues, enabling the identification of novel or rare cell types (7). This contributes to a better understanding of cell communication networks.

In recent years, several computational tools have been developed to predict cell−cell communication networks on the basis of scRNA-seq data, including celltalker (8), CellPhoneDB (9), NicheNet (10) and CellChat (11). Although all of these tools infer intercellular signalling relationships based on the expression patterns of receptors and ligands, each tool has its own distinct emphasis due to the diverse approaches to computational modelling of cell signalling networks applied (12,13). Celltalker employs a threshold-based method for inference, utilizing the expression levels of ligands and receptors exceeding a predefined threshold. CellPhoneDB adopts a cell type label permutation-based approach to infer interactions between cells. In simple terms, it begins by calculating the average expression values of ligands and receptors within their respective cell types. Then, through iterative random shuffling of cell type labels, it computes the null distribution of interactions. Finally, it uses the proportion of actual enrichment scores that exceed the computed enrichment scores as the interaction scores for ligand−receptor pairs. NicheNet is a representative network-based tool for inferring cell interactions. It utilizes a custom PageRank algorithm to measure the ability of ligand−receptor pathway interactions to predict downstream pathway targets. CellChat employs a coexpression-based strategy to incorporate signalling modulators, such as soluble agonists and antagonists, into its proprietary CellChatDB database and integrates the interaction information into signalling pathways, enabling the visualization of more complex cell−cell communication networks. In a recent study, researchers systematically compared 16 resources and 7 tools for cell−cell communication analysis (14). They integrated all of these analysis resources and tools to develop an analytical framework named LIANA. This framework aids in evaluating the consistency of various analysis tools. With the generation of increasing amounts of scRNA-seq data and the development of cell−cell communication analysis tools, it has become crucial to integrate multiple data sources for cell−cell communication analysis and construct databases that can be directly accessed and explored by researchers. Although there are existing databases containing cell−cell communication analysis results, such as the Cancer Single-cell Expression Map (CancerSCEM) (15), CellMarker 2.0 (16), SPEED (17), ABC portal (18), HTCA (19) and AgeAnno (20), these databases provide cell−cell interaction information only for individual datasets within their respective fields. They lack tools for the analysis of communication networks and the ability to compare cell−cell communication networks between different groups.

Here, we present CellCommuNet, a comprehensive data resource that explores cell−cell communication networks in human and mouse tissues under normal and disease conditions using scRNA-seq. Given the advantages of the CellChat tool in comparing datasets, generating comprehensive analysis results, and providing rich visualizations, CellCommuNet adopts CellChat as the main tool in the analysis pipeline and provides users with cell−cell communication network results in a *source cell–target cell–ligand–receptor–signalling pathway* structure. Users can freely access information about the communication strength between various cell types in the datasets, as well as the associations of these interactions with signalling pathways. To enhance the database's comprehensiveness, we incorporated results from CellPhoneDB in certain sections of the database as supplementary. CellCommuNet also offers differential analysis of cell−cell communication between normal and disease conditions, allowing users to identify which communications are altered in disease conditions. The current version of CellCommuNet includes data from over 4 300 000 cells curated from 376 single datasets, covering 82 disease types and 35 tissue types sourced from CancerSCEM (15), the Cancer Single-cell State Atlas (CancerSEA) (21), the Single Cell Expression Atlas (SCEA) (22) and the Gene Expression Omnibus (GEO) (23). Furthermore, we organized 118 comparison datasets between disease and normal samples analysed in the same scRNA-seq study. As a result, we inferred 514 463 cell−cell communication networks for each ligand−receptor pair and signalling pathway, with 329 174 networks displaying differences between disease and normal samples. In brief, CellCommuNet presents cell−cell communication analysis results in an interactive graphical format, enabling the differential analysis of communication strength between disease and normal samples. This allows users to deeply explore the transmission of intercellular information in various states. As the most comprehensive cell−cell communication network database available at present, we hope CellCommuNet will become a valuable data resource for biologists.

## Materials and methods

### Data collection and curation

The single-cell RNA-seq datasets and associated metadata information (including dataset ID, species, disease type, organ/tissue, therapeutic regime, sequencing platform, etc.) in CellCommuNet were sourced from CancerSCEM, CancerSEA, SCEA and GEO. Datasets associated with metastatic cancers and from patients treated with drug therapy were excluded. After normalization of expression profile data and quality control to remove low-quality cells, a total of 4 327 804 cells from 376 datasets across humans and mice were collected into CellCommuNet. These datasets encompass 35 tissue types and 82 disease types. Based on the metadata information of these datasets, we organized 118 comparison datasets between disease and normal samples from the same scRNA-seq study to enable the comparison of cell−cell communication between these pairs.

### Data processing pipeline

The analysis pipeline for the scRNA-seq datasets was based on Seurat (v4.1.1) (24). Key functions and parameters of used data analysis tools was shown in Supplementary Table S1. In brief, cells in each sample with RNA feature numbers (nFeature_RNA) outside the 200 to 90% maximum range were eliminated from downstream analysis. After integrating the filtered samples, we applied the SCTransform (v0.3.5) (25) function to normalize the unique molecular identifier (UMI) data. Subsequently, Harmony (v0.1.1) (26) was used to remove batch effects among samples. The ‘RunTSNE’ and ‘RunUMAP’ functions in Seurat implement the clustering algorithm for identifying clusters of distinct cell populations, and then the ‘FindAllMarkers’ function was used to identify the marker genes for each subpopulation. In cell type annotation, CellCommuNet used the ScType (27) method to determine the cell type of each cell population. To comprehensively annotate diverse tissues, we integrated CellMarker 2.0 with the marker gene reference set of ScType to expand the types of tissues annotated, e.g. breast, adipose, etc. Furthermore, we incorporated cell annotation label metadata from the original articles when available for each dataset to ensure a higher level of accuracy in our analyses.

The CellChat (v1.5.0) (11) package was used to infer and analyse intercellular communications by using mass action models based on the expression of ligands, receptors, soluble agonists and antagonists. This ligand−receptor pair (L–R pair) information was integrated into CellChatDB across human and mouse species and was also divided into three interaction patterns, i.e. secreted signalling, ECM–receptor and cell−cell contact. For each single dataset, the analysis workflow began by extracting the expression matrix and metadata from the Seurat object and then utilizing the ‘createCellChat’ function to generate a CellChat object. After conducting computations on highly variable genes and pathways, we employed the ‘computeCommunProb’ function to infer cell−cell communication probabilities. Throughout this process, we specified the parameter *‘type = ‘triMean’’* to define the method for calculating the average gene expression within each cell group, aiming to capture fewer but stronger interactions. Subsequently, we used a series of visualization functions provided by CellChat to display the results. CellCommuNet provided the strength of communications for each cell type, identified the global communication patterns to connect cell types with signalling pathways, and calculated the contribution of each signalling pathway to the roles (sender, receiver, mediator, influencer) using centrality measures from network analysis. For each comparison dataset, we firstly integrated multiple samples from different groups. Subsequently, we carried out both the Seurat workflow and the CellChat workflow analysis on each single dataset. Then, we employed the ‘liftCellChat’ function in the CellChat package to update the slots of the S4 object to merge the disease and control CellChat objects. The ‘rankNet’ function was used to compare the overall information flow of each signalling pathway. Meanwhile, the ‘netVisual_bubble’ function was utilized to identify differentially regulated signalling ligand−receptor pairs.

The CellPhoneDB (V4.0.0) (9) tool was also employed for inferring cell−cell interactions. Initially, we extracted the count matrix and cell annotation labels from the Seurat object, generating ‘count.txt’ and ‘meta.txt’ files, respectively. Subsequently, within the constructed CellPhoneDB virtual environment, we utilized the ‘statistical_analysis’ mode of the ‘method’ command to calculate cell−cell communication strength values. Given that the exported expression matrix used gene symbols as row identifiers, the ‘–counts-data’ parameter was set to ‘gene_name’. Once the ‘means.txt’ and ‘pvalues.txt’ files were created, we employed the ‘CellPhoneDB plot’ command to generate visual outputs. All resulting files were processed and organized before being incorporated into the database.

### Database construction

CellCommuNet is freely available at http://www.inbirg.com/cellcommunet/. The online database framework was constructed by Django (v2.2.5) and deployed on NGINX and uWSGI in a centOS environment. All CellCommuNet datasets are stored and managed with the MySQL (v8.0.26) server and filesystem. Front-end packages such as jQuery (v1.10.2), DataTables (v1.10.21) and Highcharts (v10.0.0) were utilized for the visual presentation of the results. Statistical analyses were performed using Python packages such as pandas (v1.4.1) and numpy (v1.23.1).

## Results

### Overview of CellCommuNet

**Figure 1.**
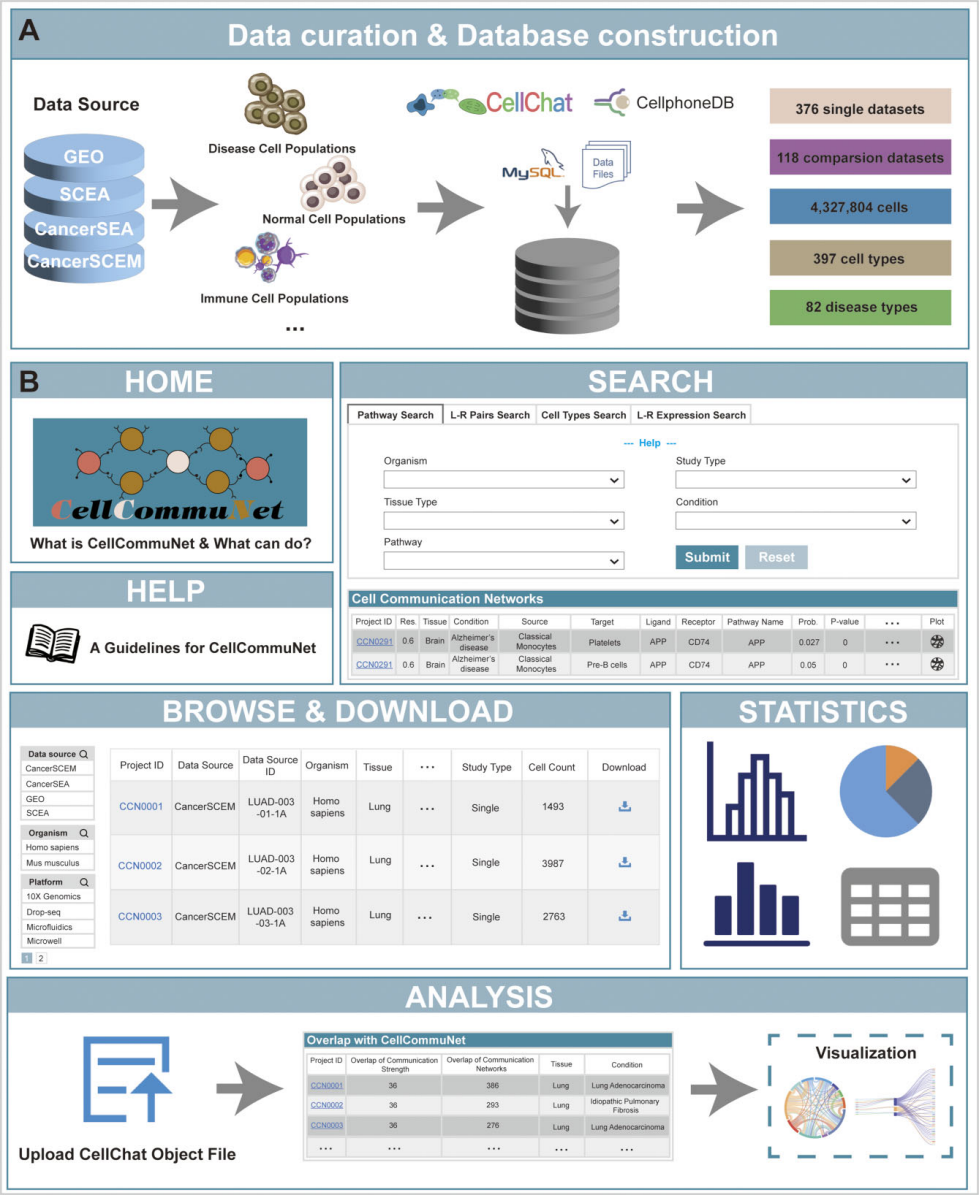
Overview of CellCommuNet. (**A**) The data sources and the workflow of the database, displaying fundamental statistical information about the data. (**B**) The main pages of CellCommuNet, including ‘HOME’, ‘SEARCH’, ‘HELP’, ‘BROWSE & DOWNLOAD’, ‘ANALYSIS’ and ‘STATISTICS’.

CellCommuNet is a database focused on the results of cell−cell communication analyses of various tissues in normal and disease conditions, providing users with a resource for exploring cell−cell interactions from multiple perspectives (Figure 1A). In the current version of CellCommuNet, the scRNA-seq profiles of 4 327 804 cells were collected from 376 datasets, including 331 human datasets and 45 mouse datasets covering 82 diseases and 35 tissue types and comprising a total of 397 nonredundant cell types including those labeled by authors in the original publications. The best represented disease types in these datasets include glioblastoma, pancreatic ductal adenocarcinoma, acute myeloid leukaemia, lung adenocarcinoma and idiopathic pulmonary fibrosis, with the top tissue types being brain, pancreas, bone marrow and lung. Based on these collected datasets, we organized 118 comparison datasets between disease and normal samples from the same scRNA-seq study. CellCommuNet constructed a cell−cell communication network consisting of the *source cell–target cell–ligand−receptor–signalling pathway* through CellChat, which is a tool that uses scRNA-seq data to quantitatively infer intercellular communication while using network analysis and pattern recognition approaches to predict how cells and signalling pathways coordinate to achieve specific functions. Currently, a total of 514 463 cell−cell communication networks of each L–R pair and each signalling pathway were inferred in CellCommuNet. The most frequent interactions were in the autocrine network between natural killer cells through different ligand receptors and signalling pathways. Among the comparison datasets, 329 174 networks with differences between the disease and healthy groups were identified, and the most commonly differential networks were in the autocrine network of natural killer cells and endothelial cells.

### Features and utilities of CellCommuNet

CellCommuNet provides a user-friendly web service including six main pages: ‘HOME’, ‘SEARCH’, ‘BROWSE & DOWNLOAD’, ‘ANALYSIS’, ‘STATISTICS’ and ‘HELP’ (Figure 1B). The ‘HOME’ page provides a brief introduction to the basic overview and functionalities of CellCommuNet. The ‘SEARCH’ page consists of four query modes, namely, pathway-based, ligand−receptor pair-based, source–target cell-based, and ligand−receptor gene expression level queries, so that users can explore cell−cell communication analysis results based on their specific research needs. On the ‘BROWSE & DOWNLOAD’ page, users can access basic information about all the collected datasets. This page provides download links for users to obtain the corresponding analysis results for the datasets. By clicking on the project ID, users can access detailed analysis results for each dataset. The ‘ANALYSIS’ page enables users to upload their own processed CellChat objects to explore and visualize the results of the cell−cell communication analysis. This function also provides a list of CellCommuNet datasets and the corresponding numbers of common nonredundant cell−cell communication networks with the user's dataset. The ‘STATISTICS’ page presents statistical information about the data available in CellCommuNet, and the ‘HELP’ page provides a detailed user guide to help users quickly get started with using CellCommuNet. The ‘SEARCH’ and ‘BROWSE & DOWNLOAD’ pages are described in detail in the sections below.

### Search function

**Figure 2.**
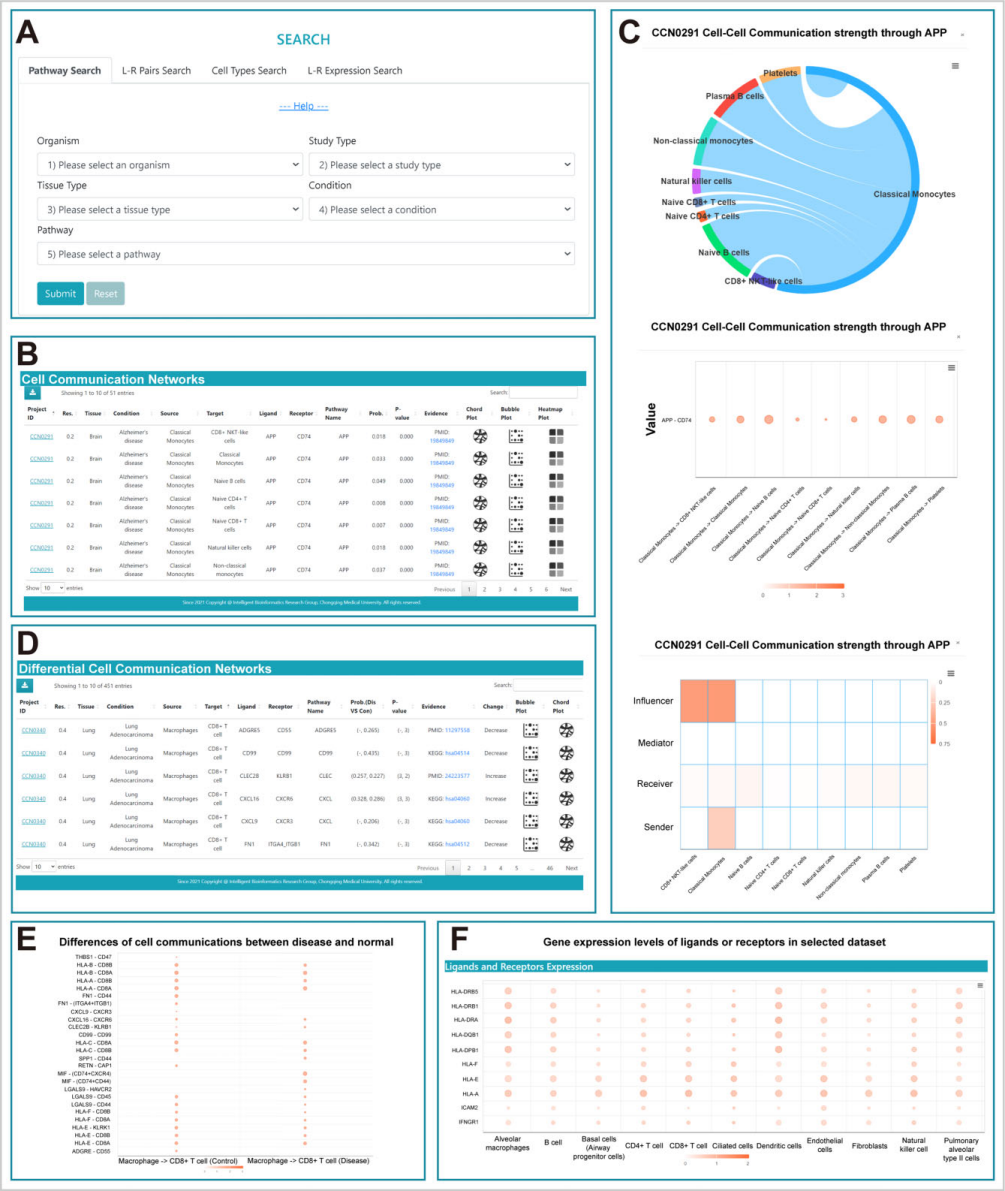
Search functions in CellCommuNet. (**A**) The *Pathway Search* tab includes five selection boxes: *Organism*, *Study Type*, *Tissue Type*, *Condition* and *Pathway*. Clicking the Help button shows/hides tutorials. (**B**) The search result table for a single dataset, containing cell−cell communication results related to the query criteria, with three visualizations provided for each communication. (**C**) The chord plot shows the communication strength between individual cells through specific pathways; the bubble plot provides specific communication information, with the size representing the probability of communication and the colour indicating the *P*-value; and the heatmap reveals the contributions of individual cell types to major roles in communication. (**D**) The search result table of a comparison dataset presents the differences in communication between disease and normal samples. *P*-value = ‘3′ means *P*-value < 0.01, *P*-value = ‘2′ means 0.01 < *P*-value < 0.05 and *P*-value = ‘1′ means *P*-value > 0.05. (**E**) Comparison of individual communication strength. (**F**) Expression of ligand or receptor genes in cell subpopulations. The size represents the percentage of cells expressing the gene, and the colour indicates the mean expression value.

Cell communication analysis based on scRNA-seq data relies on the expression levels of genes encoding ligands and receptors. The CellChat tool uses the law of mass action to infer the probability of communication between cells based on the expression of L–R pairs and various complexes and associates the communication results with signalling pathways. Thus, CellCommuNet allows users to query signalling pathways, L–R pairs, and source–target cell interactions in datasets of interest, and it provides the ability to access the expression levels of the ligand−receptor genes of interest within the datasets (Figure 2A).

The *Pathway Search* mode on the ‘SEARCH’ page allows users to query specific signalling pathways of interest. The tab currently contains five selection boxes: *Organism, Study Type, Tissue Type, Condition (*including various disease states and *‘normal’)*, and *Pathway* (Figure 2A). Users need to select the relevant information sequentially to select the dataset and finally select the pathway to query. Notably, except for *Study Type*, where users need to choose between ‘Single’ and ‘Comparison’, all other options allow selection from all available items (e.g. ‘All Organisms’). However, selecting all options may result in an excessive number of query results; therefore, we have limited the maximum number of results to 50 000. Additionally, the tab currently provides a help button that offers a brief overview of the page functionality and usage. After selecting the content of interest, users can click on the submit button to obtain the results of the analysis, wherein different *Study Type* selections correspond to different results. For the *Single* dataset option, we provide a table of the cell−cell communication results (Figure 2B), which allows users to obtain basic information about the dataset to which each communication belongs, the cell types of the source and target cells and L–R pairs, and the probability and *P*-value of communication mediated by the queried pathway. This page also provides evidence for the corresponding ligand−receptor interactions from CellChatDB. Furthermore, three visualizations are used to present the communication results (Figure 2C). The chord plot illustrates the overall strength of cell−cell communication, and the bubble plot displays the probability for each communication a by the queried signalling pathway in the corresponding dataset. The heatmap shows the major senders, receivers, mediators and influencers in the intercellular communication networks mediated by the queried pathway. Finally, users can click on the project ID to navigate to the detailed analysis results page for the dataset, which is described in the following sections. For *Comparison* datasets, in addition to the aforementioned basic dataset information, source and target cell types, L–R pairs, etc., the page presents the probabilities of communication in the disease and control groups as well as the *P*-value in a comparative format (disease vs. control), where *P*-value=’3’ means *P*-value < 0.01, ‘2′ means 0.01 < *P*-value < 0.05, and ‘1′ means *P*-value > 0.05 (Figure 2D). Additionally, according to the comparison of the probabilities, whether communication was decreased or increased in the disease condition is shown. A bubble plot is used to visualize the comparison of communication in the different samples (Figure 2E), while chord diagrams are provided to showcase the differences in communication strength between the disease and control groups in selected conditions (Supplementary Figure S1A). Similarly, clicking on the project ID enables navigation to the detailed results page.

The *L–R Pairs Search* mode and *Cell Types Search* mode on the ‘SEARCH’ page are similar to the above and allow users to query ligand−receptor pairs of interest and source or target cell types, respectively. However, these two tabs provide six selection boxes, including *Organism, Study Type, Tissue Type, Condition* (including various diseases and ‘normal’), *Ligand (*or *Source)* and *Receptor (or Target)*. Furthermore, these two modules also provide analysis results using the CellPhoneDB tool, and users can switch analysis tools to obtain the corresponding results. It is worth noting that when using the CellPhoneDB tool, species and study types are limited to a single dataset for humans (Supplementary Figure S1B). For the query results of these two modes, in the *Single* datasets, only a bubble plot is used for visualization (Supplementary Figure S1C), which is consistent with the previous description, while for the *Comparison* datasets, the visualizations are the same as described above.

The *L–R Expression Search* mode on the ‘SEARCH’ page allows users to query the expression levels of ligand−receptor genes of interest in the relevant datasets. This tab consists of six selection boxes and one input box. Users are required to use the selection boxes to determine the desired study and the resolution of cell clustering. Finally, users need to type the names of the genes they want to query in the input box, ensuring that multiple gene names are separated by semicolons. CellCommuNet then provides bubble plots displaying the expression levels of the queried genes in the corresponding datasets (Figure 2F).

One case study for a *Single* dataset is shown below, where users select Organism: ‘Homo sapiens’, Study Type: ‘Single’, Tissue Type: ‘Brain’, Condition: ‘Alzheimer's disease’ and Pathway: ‘APP’ in the *Pathway Search* mode to obtain the cell−cell communication analysis results related to Alzheimer's disease (AD) mediated by the APP signalling pathway in humans. The CCN0291 dataset in the results is derived from a study exploring the immune environment in AD (28), where cell−cell communication analysis revealed that monocytes are the primary transmitters of signals in the APP pathway and interact with other immune cells (Figure 2C). A previous study demonstrated that monocytes interact with other cells in the circulating blood, and the differential expression of critical ligand−receptors, such as APP, was observed between AD patients and treated groups (29). Consistent with this finding, the results presented by CellCommuNet suggest that monocytes may play a crucial regulatory role in the development of AD.

Another case study for a *Comparison* dataset was performed as follows: Organism: ‘Homo sapiens’, Study Type: ‘Comparison’, Tissue Type: ‘Lung’, Condition: ‘Lung Adenocarcinoma’, Source: ‘Macrophage’ and target: ‘CD8+ T cell’ in the *Cell Types* mode. CellCommuNet provides results illustrating the differences in cellular communication between lung adenocarcinoma and normal samples. The results from dataset CCN0340 demonstrate enhanced cell−cell communication mediated by SPP1 (SPP1–CD44) between macrophages and CD8+ T cells in lung adenocarcinoma (Figure 2E), a phenomenon also observed in the study by Hu *et al.* (30). This suggests that the conversion of macrophages to M2-like tumour-associated macrophages in tumour samples affects prognosis through interaction with CD8+ T cells.

### Browse & download interface

The ‘BROWSE & DOWNLOAD’ page utilizes a data table to list all the datasets that have been collected and analysed by CellCommuNet. The filter panel on the left side enables users to quickly locate studies of interest based on specific criteria. The table presents key information about each dataset, including data source, organism, tissue, cell counts, etc. In addition, CellCommuNet provides a download link for each dataset to download the expression matrix, cell annotation metadata, and cell−cell communication analysis results corresponding to the respective dataset. By clicking on the project ID, users can access a detailed results page, as described below.

### Detailed information

The dataset metadata is shown in the table on the ‘BROWSE & DOWNLOAD’ page, and more detail is available in the dataset information tab on the detailed information page, including a description of the study to which the dataset relates as well as information about the publication and the PMID link. In addition, links to data sources (CancerSCEM, CancerSEA, SCEA and GEO) have been provided. For single datasets, we provide the sample source ID, while for comparison datasets, we provide separate CellCommuNet IDs for disease and normal samples to allow users to access the corresponding datasets for each group.

**Figure 3.**
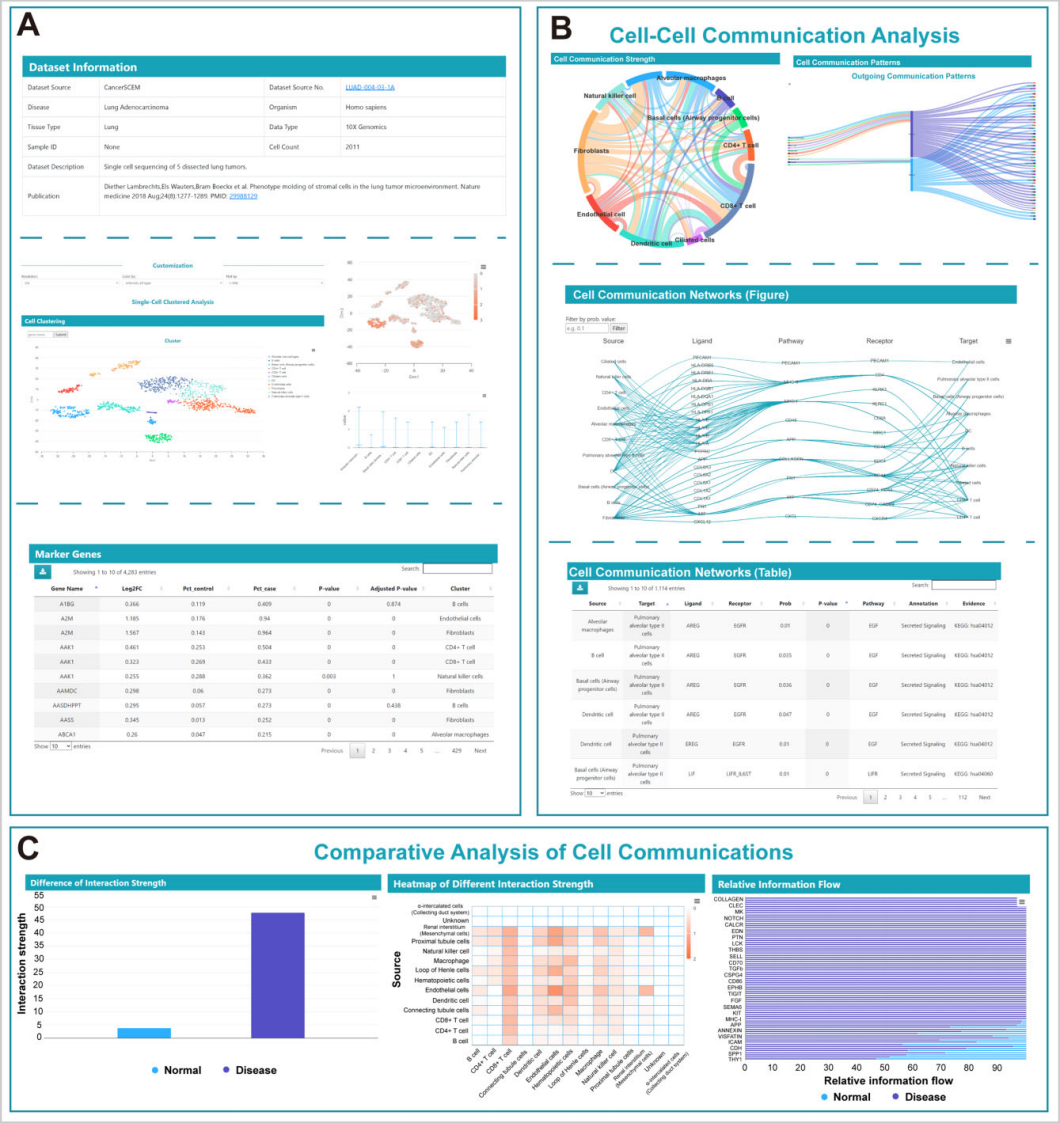
Detailed information for each dataset. (**A**) Basic information of single datasets and single-cell cluster analysis. (**B**) Cell−cell communication analysis of single datasets. The chord plot demonstrates the total communication strength of each cell population, the Sankey plot shows the communication patterns, and the cell−cell communication networks are presented using a network diagram and table. (**C**) Detailed page of the comparison datasets. The bar plot compares the total communication strength in the disease and normal states, the heatmap shows the increased or decreased communication between cell populations, and the stacked bar graphs compare the relative information flow of each pathway.

The results for single datasets are divided into two parts. The first part consists of single-cell clustering and cell annotation results. Users can customize the display of these results by modifying different parameters, including resolution (default is publication when available), cell colouring method (default is inferred cell type), and dimensionality reduction method (t-SNE or UMAP). Importantly, the choice of resolution parameter also affects the cell−cell communication results. Additionally, users can query the expression levels of specific genes. Moreover, we provide a table of marker genes for each cell subpopulation (Figure 3A). The second part presents the results of the cell−cell communication analysis (Figure 3B). A chord diagram is used to illustrate the communication strength between various cell types within this dataset. Users have the option to select and view analysis results from either CellChat or CellPhoneDB. Network analysis and pattern recognition methods are employed to predict the major signal inputs and outputs of cells, as well as how these cells coordinate with the signalling pathway. The results are visualized using Sankey diagrams. Additionally, users have the option to filter the cell−cell communication network results based on communication probability values (default displaying the top 50% highest communication strength). Finally, we provide a table of cell−cell communication analysis results at the selected resolution for further research and analysis by the users.

For comparison datasets, CellCommuNet focuses on presenting the results of differential analysis of cell−cell communication (Figure 3C). Similarly, users can customize the parameters to view the results at different resolutions. We first utilize bar plots to demonstrate the differences in the overall strength of cell−cell communication under different conditions. Heatmaps are employed to visualize the differences in communication between individual cells. Additionally, we display the differences in information flow for various signalling pathways. In CellChat, the information flow for a given signalling pathway is defined as the sum of the communication probability among all pairs of cell groups in the inferred communication network, and the relative information flow is visualized using a stacked bar plot. Similarly, we provide a table of cell−cell communication differences at the selected resolution for users to explore further (Supplementary Figure S1D).

### Case studies

#### Case study for a Single dataset

The CCN0001 dataset, sourced from CancerSCEM, includes data from lung adenocarcinoma. A total of 1493 cells were detected in this dataset, and at the default resolution, 11 distinct cell types were identified, including basal cells, ciliated cells, dendritic cells, endothelial cells, fibroblasts, pulmonary alveolar type II cells, and a range of immune cells. From the results of cell−cell communication strength, it was evident that fibroblasts played a significant role as the primary signal senders in the cell−cell communication network and interacted with other cells (Figure 3B). This observation is likely attributable to the production of various growth factors and proinflammatory factors by fibroblasts, which contribute to the recruitment of immunosuppressive cells (31). Additionally, the Sankey diagram highlighted that fibroblasts primarily engage in an outgoing communication pattern, sharing a similar pattern with endothelial cells and pulmonary alveolar type II cells. The main signalling pathways involved in this pattern include FN1, CXCL and others.

#### Case study for a comparison dataset

CCN0421 is a comparison dataset associated with clear cell renal cell carcinoma (ccRCC) sourced from the GEO database (Figure 3C). The CellCommuNet IDs for the disease and normal datasets were CCN0331 and CCN0329, respectively. A total of 14 140 cells were compared, and from the bar plot, it can be observed that cell−cell communication is more active in ccRCC. The heatmap results indicated that endothelial cells exhibit increased interaction with other cells in terms of both outgoing and incoming signals compared to that in normal samples. This finding suggests that endothelial cells may play a regulatory role in tumour development (32,33). In terms of information flow, almost all tumour-related signalling pathways show increased activation compared with that in normal samples, including the VEGF, FN1 and FGF pathways. These results highlight the heightened cell−cell communication activity and the activation of specific signalling pathways in ccRCC, potentially contributing to the understanding of tumour progression and identifying potential therapeutic targets.

## Discussion and future directions

CellCommuNet is a user-friendly, interactive database that provides cell−cell communication analysis results based on scRNA-seq data from the CancerSCEM, CancerSEA, SCEA and GEO databases, focusing on human and mouse data and diseases. In CellCommuNet, users can freely explore the interactions between cells in various disease states. Research on cell communication has greatly benefited from advances in transcriptomics, especially single-cell transcriptomics, and many studies now focus on signalling pathways that mediate cell−cell communication, relevant ligand−receptor pairs, and cell types in tissue- or organ-involved interactions (34–37). Therefore, CellCommuNet provides four search modes on the search page, allowing users to explore specific signalling pathways, ligand−receptor pairs, cell types and the expression level of ligand or receptor genes. By using the provided keywords in the selection boxes, users can access the corresponding cell−cell communication analysis results, providing data to support subsequent experimental validation. Furthermore, since cell communication plays a crucial role in maintaining homeostasis in living organisms, it is expected that alterations will occur in cell communication during disease states (38,39). CellCommuNet includes 118 comparative datasets of disease and normal samples to provide differential cell−cell communication analyses, aiding further exploration of disease progression. Another highlight of CellCommuNet is its utilization of the nonnegative matrix factorization algorithm (40) and network analysis based on CellChat, which provides cell−cell communication patterns to users and integrates them with signalling pathways, helping researchers explore complex cell−cell communication networks. Existing databases do not offer such comprehensive analysis. For example, the ABC portal database utilizes CellPhoneDB 2.0 to perform cell−cell communication analysis specifically for blood and immune cells, enhancing the understanding of haematopoiesis and blood/immune diseases, but lacks integration of signalling pathways (18). Similarly, the SPEED database utilizes CellChat for cell−cell communication analysis, but it is focused on pan−species maps and has limited datasets for human and mouse diseases; moreover, it does not provide comparative analysis of cell−cell communication in different states (17). Therefore, to our knowledge, CellCommuNet is the only comprehensive database focusing on cell−cell communication networks in humans and mice under both disease and normal states. In the future, we will continue to maintain and update CellCommuNet. New data can be seamlessly added to CellCommuNet through our standardized analysis pipeline. We plan to conduct reviews of four data sources every six months to incorporate new datasets.

However, there are still areas for improvement: (i) cell communication is not mediated solely by signalling molecules, and the spatial information of cells is also crucial (41,42). Integrating single-cell spatial omics data to explore cell communication will be one of our future directions. (ii) CellCommuNet is currently primarily based on CellChat software and relies on CellChatDB as a reference database, although we have incorporated certain results from analyses conducted using CellPhoneDB. However, we recognize that achieving comprehensive coverage of the database results remains a challenge. In the future, we will strive to collect and incorporate data for more signalling molecules and integrate more cell communication inference tools into our database to provide more comprehensive cell communication network information. (iii) Misannotations of cell types in scRNA-seq analysis will generate incorrect cell−cell communication network information. In the current version, we provide two cell annotation approaches: one from the original publication and the other from ScType. In the future, we will integrate more cell type markers to improve the annotation of cell types. (iv) The current focus of cell communication analysis tools primarily centres on detecting signals emitted by cells themselves, often overlooking external signals originating from the surrounding environment (43). Recognizing the significance of these external signals in influencing cell activities and fates, we plan future enhancements in CellCommuNet. This involves identifying external signals within the cell communication network and exploring the ligand−receptor pairs that activate transcription factors and downstream target genes. By doing so, we aim to enrich the downstream information within the cell communication network, thereby further improving the comprehensiveness of CellCommuNet. We believe that CellCommuNet will contribute to researchers' understanding of cell−cell communication and provide insights into the mechanisms of disease development.

## Supplementary Material

gkad906_Supplemental_Files

## Data Availability

All data, including the results and metadata, are available at http://www.inbirg.com/cellcommunet/ without login requirement, and the visualization results can be downloaded in PNG, JPEG, PDF and SVG formats.
