# Supplementary material for: CellCommuNet: an atlas of cell–cell communication networks from single-cell RNA sequencing of human and mouse tissues in normal and disease states
Source: Nucleic Acids Res. 2023 Oct 18;52(D1):D597–606. doi: 10.1093/nar/gkad906 (PMC10767892; doi:10.1093/nar/gkad906)
Supplement: gkad906_Supplemental_Files [file gkad906_supplemental_files.pdf]

Supplementary information

CellCommuNet: an atlas of cell – cell communication networks from single-cell RNA sequencing of human and mouse tissues in normal and disease states

Supplementary Table S1. Key functions and parameters of used data analysis tools.

Supplementary Figure S1. CellCommuNet search functions and detail page. (A) The differences in communication strength between disease and control groups. Positive values indicate upregulation of communication in the disease group, while negative values indicate downregulation in the disease group; (B) The *L-R Pairs Search* tab. Users can query results under different analysis tools through the selector in the red box; (C) Result table of L-R pairs search and cell types search; (D) Differences in communication networks.

Supplementary Table S1. Key functions and parameters of data analysis workflow

Software	Function	Parameter	Value	Description
Seurat (v4.1.1)	SCTransform (v0.3.5)	do.scale	FALSE	Whether to scale residuals to have unit variance
		do.center	TRUE	Whether to centre residuals to have a mean of zero
	RunTSNE/RunUMAP	reduction	harmony	Which dimensional reduction to use (t-SNE or UMAP)
	FindClusters	resolution	0.2/0.4/0.6 /0.8/1	Value of the resolution parameter
	FindAllMarkers	assay	RNA	Assay to use in differential expression testing
		slot	data	Data for differential analysis
		only.pos	TRUE	Only return positive markers
		logfc.threshold	0.25	DEG cut-off
		test.use	wilcox	Identifies differentially expressed genes between two groups of cells using a Wilcoxon Rank Sum test
Harmony (v0.1.1)	RunHarmony	group.by.vars	orig.ident	Remove batch effects between samples (orig.ident)
		assay.use	SCT	Which assay to harmonize with
		max.iter.harmony	10	Maximum number of rounds to run Harmony
		lambda	1	Ridge regression penalty parameter
CellChat (v1.5.0)	createCellChat	group.by	celltype	Group by cell type
	computeCommunProb	type	triMean	Methods for computing the average gene expression per cell group.
		raw.use	TRUE	Use raw data instead of data mapped to the PPI network
	filterCommunication	min.cells	10	The minimum number of cells required in each cell group for cell–cell communication

	computeCommunProbPathway	thresh	0.05	Threshold of the p-value for determining significant interaction
	aggregateNet	thresh	0.05	Threshold of the p-value for determining significant interactions
	netAnalysis_computeCentrality	slot.name	netP	Slot name of the object used to compute the centrality measures of the signalling networks
		thresh	0.05	Threshold of the p-value for determining significant interactions
	identifyCommunicationPatterns	slot.name	netP	Slot name of the object used to compute the centrality measures of the signalling networks
		pattern	outgoing/incoming	Identify outgoing or incoming signal patterns
	liftCellChat	group.new	group.new.control/group.new.disease	Unify cell annotation labels for two datasets
	mergeCellChat	cell.prefix	TRUE	Whether prefix cell names
		merge.data	FALSE	Only merge data for signalling genes (rather than all genes)
	rankNet	mode	comparison	Use the comparison mode to compare the overall information flow of two datasets
		stacked	TRUE	Return the stacked bar plot
		return.data	TRUE	Return the data.frame consisting of the calculated information flow of each signalling pathway or L-R pair
		measure	count/weight	Compare the number of interactions or the total interaction strength

CellPhoneDB (V4.0)	method statistical_analysis	-	meta.txt	Metadata of scRNA-seq dataset
		-	count.txt	Expression matrix of scRNA-seq dataset
		--counts-data	gene_name	Type of gene identifiers in the counts data
		--iterations	1000	Number of iterations for the statistical analysis
		--pvalue	0.05	P-value threshold

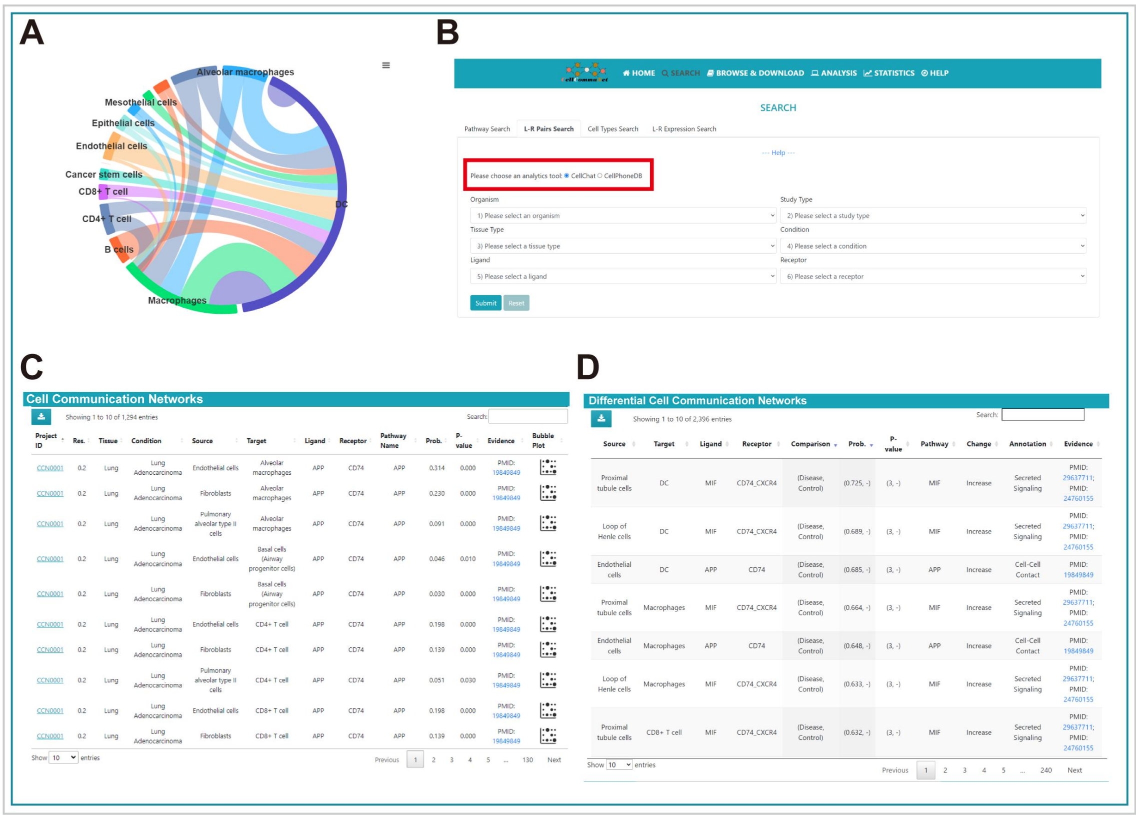

Supplementary Figure S1. CellCommuNet search functions and detail page. (A) The differences in communication strength between disease and control groups. Positive values indicate upregulation of communication in the disease group, while negative values indicate downregulation in the disease group. (B) The *L-R Pairs Search* tab. Users can query results under different analysis tools through the selector in the red box. (C) Result table of L-R pairs search and cell types search. (D) Differences in communication networks.
